# The Impact of Balloon Pulmonary Angioplasty (BPA) on Changes in the Liver Fibrosis Index (FIB-4 Index) in Patients with Chronic Thromboembolic Pulmonary Hypertension (CTEPH)

**DOI:** 10.3390/jcm15187251

**Published:** 2026-09-18

**Authors:** Ilona Skoczylas, Jacek Piegza, Marcin Waligóra, Maciej Skoczylas, Michał Wróbel, Mateusz Tajstra, Mariusz Gąsior

**Affiliations:** 1 3rd Department of Cardiology, Faculty of Medical Sciences in Zabrze, Silesian Centre for Heart Diseases, Medical University of Silesia, 41-800 Zabrze, Poland; j.piegza@sccs.pl (J.P.); wrm@o2.pl (M.W.); 2 Department of Cardiac and Vascular Diseases, John Paul II Hospital in Krakow, 31-202 Krakow, Poland; marcin.waligora@uj.edu.pl; 3 Pulmonary Circulation Centre, Department of Cardiac and Vascular Diseases, Faculty of Medicine, Jagiellonian University Medical College, 31-008 Krakow, Poland; 4 Center for Innovative Medical Education, Department of Medical Education, Faculty of Medicine, Jagiellonian University Medical College, 30-688 Krakow, Poland; 5 Department of Internal Medicine & Cardiology, Medical University of Warsaw, 02-005 Warsaw, Poland; s085305@student.wum.edu.pl; 6 3rd Department of Cardiology, Faculty of Medical Sciences in Zabrze, Medical University of Silesia, 40-055 Katowice, Poland; m.tajstra@sccs.pl (M.T.); m.gasior@sccs.pl (M.G.)

**Keywords:** chronic thromboembolic pulmonary hypertension (CTEPH), balloon pulmonary angioplasty (BPA), fibrosis-4 index (FIB-4 index), hepatic congestion, right ventricular function

## Abstract

**Background:** Progressive right ventricular dysfunction and elevated right atrial pressure, commonly observed in patients with Chronic Thromboembolic Pulmonary Hypertension (CTEPH), may lead to systemic venous congestion, including hepatic congestion. The liver fibrosis index (FIB-4) has been proposed as a non-invasive marker of hepatic congestion and fibrosis. Data and evidence regarding the prevalence and clinical significance of elevated FIB-4 index values in patients with CTEPH and/or right-sided heart failure secondary to pulmonary hypertension (PH) are limited. Furthermore, it remains unclear whether interventional treatment with balloon pulmonary angioplasty (BPA), which improves right ventricular (RV) function and pulmonary hemodynamics and lowers pressure in right atrium and hepatic veins, also alleviates hepatic congestion and imitatively decreases the FIB-4 index. **Aim:** To investigate whether BPA-induced improvement in pulmonary hemodynamics and right ventricular function is accompanied by changes in the FIB-4 index in patients with CTEPH. **Methods:** This single-centre, retrospective analysis included 36 consecutive patients with CTEPH who underwent BPA and were followed at a tertiary PH centre between June 2020 and November 2025. All patients underwent comprehensive baseline evaluation prior to the first BPA session and repeated assessment after completion of the staged BPA treatment. Clinical evaluation included WHO functional class (WHO-FC) and the 6 min walk distance (6MWD). Right heart catheterization (RHC) was performed according to standards. Laboratory testing included N-terminal pro-brain natriuretic peptide (NT-proBNP), creatinine, aminotransferases (ALT, AST), complete blood count, and the fibrosis-4 (FIB-4) index. FIB-4 was classified into three categories: <1.3 indicating low FIB-4 category, 1.3–2.67 representing an indeterminate or borderline range, and >2.67 indicating high FIB-4 category. Change in FIB-4 status was assessed categorically. Improvement was defined as a downward shift of ≥1 category between baseline and follow-up; stability was defined as no categorical change; and worsening as an upward shift of ≥1 category. **Results:** Among the 36 patients in whom BPA treatment was initiated, 31 completed the planned BPA sessions, underwent the final assessment, and were eligible for the current analysis. This resulted in a median decrease in mRAP of −3 (−5 to −1) mmHg, in mPAP of −9 (−13 to −1) mmHg, and in PVR of −1.7 (−3.9 to −0.3) WU, accompanied by an increase in CI of 0.24 (−0.15 to 0.71) L/min/m^2^ and a reduction in NT-proBNP of −317 (−1553 to −30) pg/mL. In parallel, FIB-4 decreased by a median of −0.20 (−0.50 to −0.07). At baseline, 7 patients had low FIB-4 values (<1.3), 16 had indeterminate values (1.3–2.67), and 8 had high FIB-4 values (>2.67). All patients with baseline FIB-4 <1.3 remained in the low category. Among the 24 patients with baseline FIB-4 ≥1.3, 8 (33.3%; exact 95% CI 15.6–55.3%) improved by one category, 14 (58.3%) remained stable, and 2 (8.3%) worsened (exact two-sided sign test among patients with categorical change, *p* = 0.109). In the exploratory binary analysis of improvement versus no improvement, with stable and worsening cases classified as no improvement, the exact one-sided McNemar test yielded *p* = 0.0039. In an exploratory multivariate linear regression analysis, only the change in NT-proBNP was associated with a concurrent change in FIB-4 (*p* = 0.0375). **Conclusions:** In our study, the FIB-4 index was elevated in 77.4% of patients with CTEPH. BPA is an effective procedure that markedly improves hemodynamics and alleviates right heart failure. In addition, FIB-4 category in every third patient who had elevated FIB-4 decreased following BPA. A reduction in NT-proBNP, a marker of right ventricular dysfunction, was the only variable associated with concurrent FIB-4 change.

## 1. Introduction

Chronic thromboembolic pulmonary hypertension (CTEPH) is characterized by persistent precapillary pulmonary hypertension (PH) associated with elevated right ventricular (RV) and right atrial pressures, ultimately leading to progressive right heart failure and premature death [1,2]. Most patients with CTEPH had prior acute pulmonary embolism [1] and CTEPH results from unresolved thromboembolic obstruction of the pulmonary arteries combined with secondary remodelling of the pulmonary vasculature. This remodelling process is driven by abnormal angiogenesis, impaired fibrinolysis, and endothelial dysfunction [3].

According to the current European Society of Cardiology/European Respiratory Society (ESC/ERS) guidelines for the diagnosis and treatment of pulmonary hypertension, therapeutic options for patients with CTEPH include pulmonary endarterectomy (PEA) as the treatment of choice, balloon pulmonary angioplasty (BPA) for patients who are ineligible for surgery, and targeted pharmacotherapy [2]. The beneficial effects of balloon pulmonary angioplasty on survival, exercise capacity, World Health Organization functional class, pulmonary hemodynamics, and N-terminal pro-B-type natriuretic peptide (NT-proBNP) levels have been consistently demonstrated and are well documented in the literature [1,3,4,5,6,7,8,9,10,11,12].

Progressive right ventricular dysfunction and elevated right atrial pressure, commonly observed in patients with CTEPH, may lead to systemic venous congestion, including hepatic congestion. The liver is one of the first organs impacted by RV failure from pulmonary hypertension (PH). RV volume and pressure overload can lead to congestive hepatopathy [13,14,15,16]. Chronic liver congestion can result in biochemical abnormalities and progressive liver fibrosis [13]; however, its prevalence and clinical relevance in this patient population remain poorly characterized. The liver fibrosis index (FIB-4) has been proposed as a non-invasive marker of hepatic fibrosis [17,18,19,20]. Elevated FIB-4 index values have been associated with a higher prevalence of heart failure [21,22], and with an increased risk of mortality in acute heart failure [23,24,25] as well as in stable chronic heart failure of various aetiology [26,27].

Nevertheless, data and evidence regarding the prevalence and clinical significance of elevated FIB-4 index values in patients with CTEPH and/or right-sided heart failure secondary to pulmonary hypertension are limited. Furthermore, it remains unclear whether interventional treatment with balloon pulmonary angioplasty (BPA), which improves pulmonary hemodynamics and right ventricular function [4,5,7,8] also alleviates hepatic congestion and improves non-invasive markers of liver fibrosis, and whether such changes correlate with clinical and hemodynamic outcomes.

Therefore, in our study we aimed to investigate whether BPA-induced improvement in pulmonary hemodynamics and right ventricular function is accompanied by changes in the FIB-4 index, a non-invasive marker of hepatic fibrosis, in patients with CTEPH.

## 2. Methods

### 2.1. Study Population

This single-centre, retrospective analysis included consecutive patients with CTEPH diagnosed according to guidelines [2] who underwent balloon pulmonary angioplasty (BPA) and were followed at a tertiary PH centre between June 2020 and November 2025. All patients were evaluated for operability by the multidisciplinary CTEPH team. Only patients who completed the planned BPA sessions and underwent the final reassessment were eligible for analysis. Clinical characteristics, medical history, right heart catheterization data, laboratory parameters, comorbidities, and information on background therapy were collected from medical records.

#### 2.1.1. Clinical and Hemodynamic Assessment

All patients underwent comprehensive baseline evaluation prior to the first BPA session and repeated assessment after completion of the staged BPA treatment. Clinical evaluation included WHO functional class (WHO-FC) and the 6 min walk distance (6MWD). Right heart catheterization was performed according to standards [28,29] and included mean right atrial pressure (mRAP), mean pulmonary artery pressure (mPAP), pulmonary artery wedge pressure (PAWP), cardiac index (CI), stroke volume index (SVI), pulmonary vascular resistance (PVR), and mixed venous oxygen saturation (SvO2). Laboratory testing included N-terminal pro-brain natriuretic peptide (NT-proBNP), creatinine, aminotransferases (ALT, AST), complete blood count, and the fibrosis-4 (FIB-4) index. FIB-4 was classified into three categories: <1.3 indicating low FIB-4 category, 1.3–2.67 representing an indeterminate or borderline range, and >2.67 indicating high FIB-4 category. The cut-off values used in our study (<1.3, 1.3–2.67, and >2.67) were selected based on the originally validated and widely used FIB-4 risk stratification thresholds [30]. Change in FIB-4 status was assessed categorically. Improvement was defined as a downward shift of ≥1 category between baseline and follow-up; stability was defined as no categorical change; and worsening as an upward shift of ≥1 category. In an analysis restricted to patients with a categorical change, improvement versus worsening was assessed using an exact two-sided sign test. In an exploratory analysis of improvement versus no improvement among patients with baseline FIB-4 ≥ 1.3, stable and worsening cases were classified as no improvement, and an exact one-sided McNemar test was used. The FIB-4 formula used in this study was as follows.

$$FIB-4=\frac{age\;\left[years\right]\times AST\;[\frac{U}{L}]}{platelet\;count\;\left[10^{9}\right]\times \sqrt{ALT\;[\frac{U}{L}]}}$$

Study participants had no documented chronic liver disease, including chronic hepatitis B or C infection, metabolic dysfunction-associated steatotic liver disease (MASLD)/metabolic dysfunction-associated steatohepatitis (MASH), or other known chronic hepatic disorders. Two patients reported regular alcohol consumption; however, neither had a documented diagnosis of alcohol-related liver disease.

#### 2.1.2. Balloon Pulmonary Angioplasty Procedure

BPA was performed in staged sessions following current expert recommendations [2,4,7,8,9]. Target lesions were identified based on selective pulmonary angiography. Interventions were performed using standard balloon catheters, with gradual vessel preparation and avoidance of aggressive balloon oversizing. Periprocedural parameters and screening complications were systematically recorded. The median follow-up interval after the final BPA procedure was 3 months.

### 2.2. Statistical Analysis

No formal a priori sample-size or power calculation was performed, as this was a retrospective analysis including all consecutive eligible patients. Exact 95% confidence intervals for proportions were calculated using the Clopper–Pearson method. Given the limited sample size, the multivariable regression analysis was considered exploratory.

Continuous variables are presented as medians with interquartile ranges (IQR). Categorical variables are presented as counts and percentages. Changes between pre- and post-BPA values were assessed using the Wilcoxon signed-rank test for paired continuous variables. Changes in WHO-FC distribution were evaluated using Bowker’s test for symmetry. Additionally, dichotomized paired categorical variables were analysed using McNemar’s test. For FIB-4, categorical improvement was additionally evaluated in an exploratory exact one-sided directional analysis. Transitions across the FIB-4 threshold of 1.3 (<1.3 vs. ≥1.3) were evaluated using the exact two-sided McNemar test. Transitions between FIB-4 categories were visualized using a Sankey diagram generated with the SankeyMATIC tool (www.sankeymatic.com; USA). Unless otherwise specified, statistical tests were two-tailed.

An exploratory multivariable linear regression analysis was performed with ΔFIB-4 as the dependent variable. The following candidate variables were evaluated based on their clinical relevance to right-heart function and hemodynamic response: ΔNT-proBNP, ΔCI, ΔmPAP, ΔPVR, and ΔmRAP. Variables with a univariable *p* value < 0.10 were eligible for inclusion in the multivariable model. Analyses were performed using Statistica 13.3 (TIBCO Software Inc.; San Ramon, CA, USA).

### 2.3. Ethical Considerations

The study was conducted in accordance with the Declaration of Helsinki and Good Clinical Practice guidelines. The study protocol was reviewed and approved by the Institutional Bioethics Committee No: BNW/NWN/0052/KB/30/26.

## 3. Results

Among the 36 patients in whom BPA treatment was initiated, 31 had completed paired pre- and post-BPA assessments and were eligible for the current analysis. In the remaining five patients, post-BPA reassessment, including right heart catheterization and other follow-up investigations, was not yet available. Among them, 25 patients (80.6%) had a history of pulmonary embolism (PE), including 3 (12.0%) with low-risk PE, 10 (40.0%) with intermediate-low risk, 10 (40.0%) with intermediate-high risk, and 2 (8.0%) classified as high risk. Five patients experienced recurrent PE, and one of them had two recurrences. Among patients with a history of pulmonary embolism, the median calendar-year interval between the index PE and initiation of riociguat was 2 years (IQR 1–4.5 years), the median interval from diagnostic RHC or riociguat initiation to the first BPA session was 145 days (IQR 113–236 days) corresponding to approximately 4.8 months (IQR 3.7–7.7 months) and the median interval from the first to the final BPA session was 516 days (IQR 173–878), corresponding to approximately 17.0 months (IQR 5.7–28.8 months). The detailed clinical characteristics are presented in Table 1.

Among the 31 patients, five (16.1%) were scheduled for BPA due to persistent CTEPH after PEA; 12 (38.7%) had lesions inaccessible for surgery; 10 (32.3%) were technically operable but declined surgery; and four (12.9%) were technically operable but were ultimately disqualified from PEA because of an unfavourable risk-benefit ratio. Twenty-eight (90.3%) patients received riociguat and one (3.2%) received sildenafil, and two (6.5%) received no targeted PH therapy as background treatment. Among the studied cohort, all patients received anticoagulant therapy. Eighteen patients (58%) were treated with apixaban, seven (22.5%) with rivaroxaban, two (6.5%) with dabigatran, and four (13.0%) with a vitamin K antagonist (warfarin). These patients underwent a median of 3 (2–4) BPA sessions, during which 17 (10–24) lesions were dilated.

BPA procedures were safe and uneventful except for two patients: one experienced transient haemoptysis-associated with a minor pulmonary vascular wire injury, while another developed transient cough during the procedure, without further sequelae. No patient died during either the in-hospital observation period or the 1-year follow-up.

**Figure 1:**
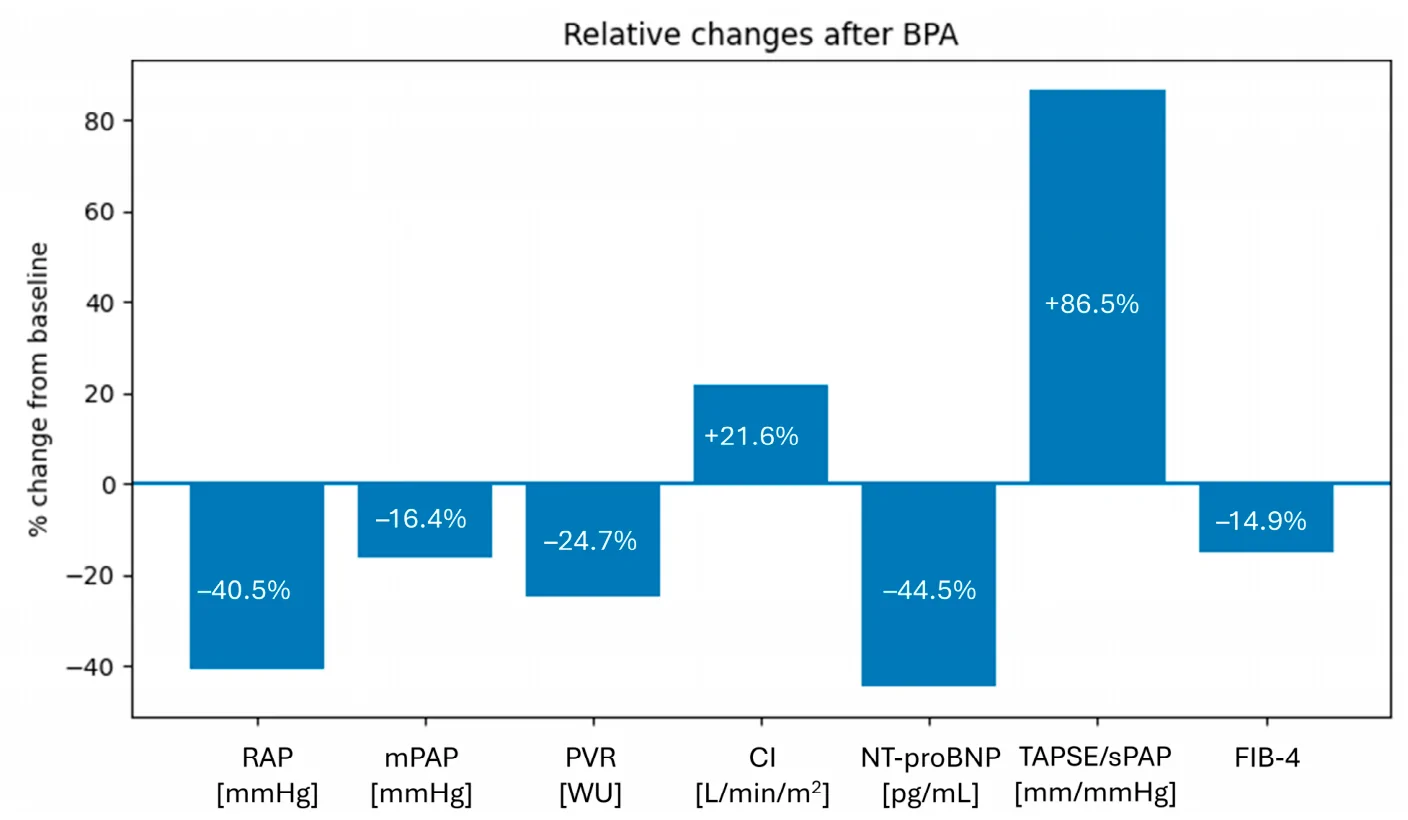
Relative changes in hemodynamic, biomarker and hepatic fibrosis parameters after BPA. RAP—right atrial pressure, mPAP—mean pulmonary artery pressure, PVR—Pulmonary Vascular Resistance, CI—cardiac index; TAPSE/sPAP—Tricuspid Annular Plane Systolic Excursion/Systolic Pulmonary Artery Pressure.

This resulted in a median decrease in m RAP of −3 (−5 to −1) mmHg, in m PAP of −9 (−13 to −1) mmHg, and in PVR of −1.7 (−3.9 to −0.3) WU, accompanied by an increase in CI of 0.24 (−0.15 to 0.71) L/min/m^2^ and a reduction in NT-proBNP of −317 (−1553 to −30) pg/mL. In parallel, FIB-4 decreased by a median of −0.20 (−0.50 to −0.07). A post hoc mathematical decomposition of the FIB-4 formula indicated that the change in platelet count was the dominant contributor to the observed change in FIB-4. When the absolute magnitude of the contribution of each FIB-4 component was normalized to 100%, platelet count accounted for approximately 53% of the total component-level change, followed by AST (35%), age (6.6%), and ALT (5.5%). The detailed effects of BPA on clinical, hemodynamic, and laboratory parameters are presented in Table 2 and depicted in Figure 1.

**Figure 2:**
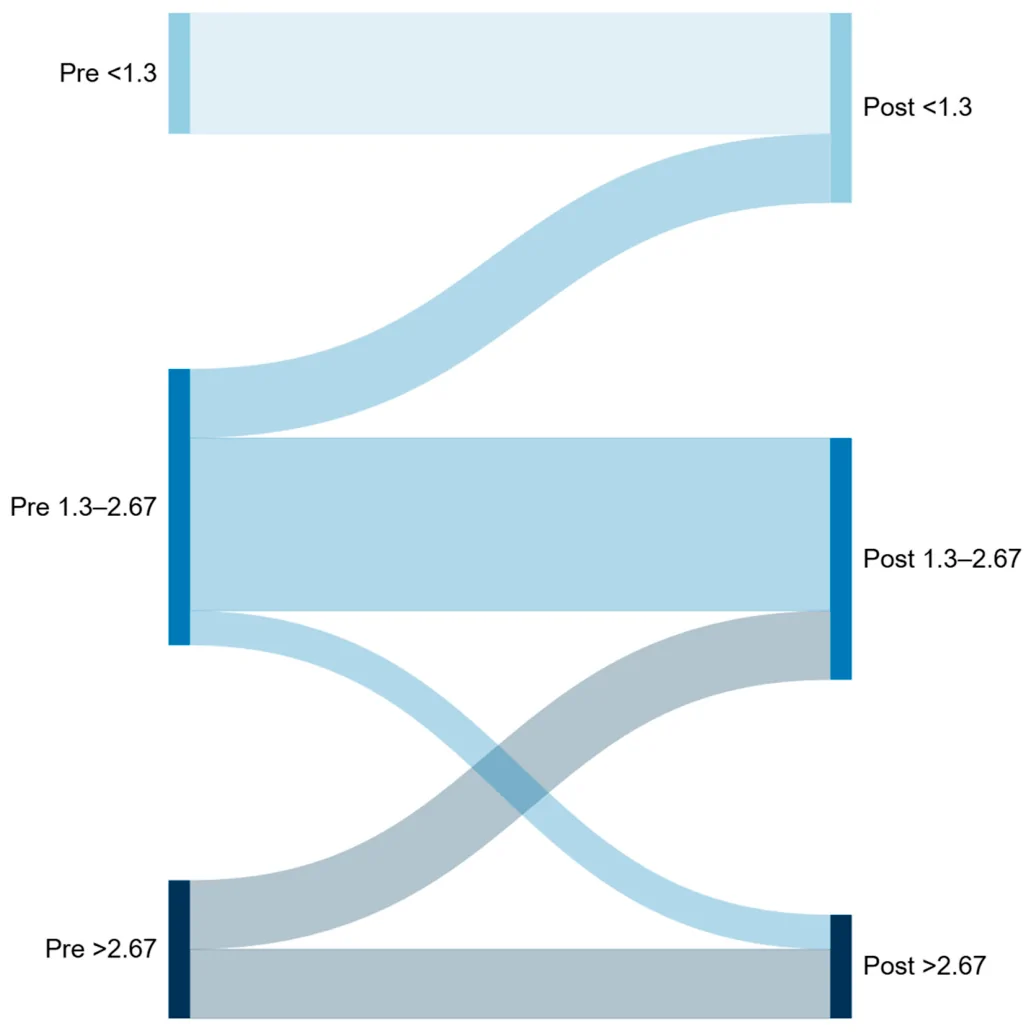
Sankey diagram illustrating transitions between FIB-4 categories before and after balloon pulmonary angioplasty. Among the 24 patients with baseline FIB-4 ≥ 1.3, 8 (33.3%; exact 95% CI 15.6–55.3%) improved, 14 (58.3%) remained stable, and 2 (8.3%) worsened (exact two-sided sign test among patients with categorical change, *p* = 0.109). In an exploratory analysis of improvement versus no improvement, with stable and worsening cases classified as no improvement, the exact one-sided McNemar test yielded *p* = 0.0039. The width of each flow is proportional to the number of patients moving between strata. FIB-4 categories were defined as <1.3 (low FIB-4 category), 1.3–2.67 (intermediate FIB-4 category), and >2.67 (high FIB-4 category).

At baseline, 7 patients had low FIB-4 values (<1.3), 16 had indeterminate values (1.3–2.67), and 8 had high FIB-4 values (>2.67). All patients with baseline FIB-4 < 1.3 remained in the low category. Among the 24 patients with baseline FIB-4 ≥ 1.3, 8 (33.3%; exact 95% CI 15.6–55.3%) improved by one category following BPA, 14 (58.3%) remained stable, and 2 (8.3%) worsened (exact two-sided sign test among patients with categorical change, *p* = 0.109). In an exploratory analysis of improvement versus no improvement (with stable and worsening cases classified as no improvement), the exact one-sided McNemar test yielded *p* = 0.0039. Four patients (16.7%) transitioned from FIB-4 ≥ 1.3 to <1.3, whereas no patient transitioned in the opposite direction (exact two-sided McNemar *p* = 0.125). Transitions are depicted in Figure 2.

In an exploratory multivariable linear regression analysis, change in NT-proBNP (ΔNT-proBNP) was the only variable associated with concurrent change in FIB-4 (β = 0.000173, 95% CI 0.000010–0.000336; *p* = 0.038) (Central Illustration—Graphical Abstract).

## 4. Discussion

To our knowledge, this is the first study to demonstrate an association between improvement in right-sided hemodynamics following BPA and changes in FIB-4, including improvement in FIB-4 category in patients with CTEPH.

### 4.1. FIB-4 in Heart Failure

The FIB-4 index, originally developed as a non-invasive tool for the assessment of liver fibrosis based on age, serum aminotransferase levels (ALT and AST) and platelet count, has recently gained increasing attention as a potential biomarker in cardiovascular diseases [21,22,27], including acute and chronic heart failure, tricuspid regurgitation or pulmonary arterial hypertension (PAH) [23,24,25,26,31,32,33,34,35,36]. Multiple clinical studies have demonstrated that the FIB-4 index is significantly associated with adverse outcomes in patients with heart failure (HF) [24]. In most cases, these outcomes included all-cause mortality and cardiovascular mortality [22,23,26,27]. In acute and chronic HF populations, higher FIB-4 scores are linked to elevated all-cause mortality, HF rehospitalization, and composite adverse cardiac events, while the FIB-4 reduction during hospitalization was associated with better prognoses. In a study including 877 patients (age 74.9 ± 12.0 years; 58% male) hospitalized with acute heart failure, the primary outcome was a composite of all-cause death or heart failure rehospitalization within 180 days. The median FIB-4 reduction was 14.7% (interquartile range—7.8–34.9%). The primary outcome was observed in 79 (27.0%), 63 (21.6%), and 41 (14.0%) patients in the low, middle, and high FIB-4 reduction groups, respectively (*p* = 0.001). Adjusted Cox proportional-hazards analysis revealed that the middle and low FIB-4 reduction groups were associated with the primary outcome, independent of the pre-existing risk model including baseline FIB-4 ([high vs. middle] hazard ratio [HR]: 1.70, 95% confidence interval [CI]: 1.10–2.63, *p* = 0.017; [high vs. low] HR: 2.16, 95% CI 1.41–3.32, *p* < 0.001). FIB-4 reduction provided additional prognostic value to the baseline model, including well-known prognostic factors ([continuous net reclassification improvement] 0.304; 95% CI 0.139–0.464; *p* < 0.001; [integrated discrimination improvement] 0.011; 95% CI 0.004–0.017; *p* = 0.001) [25]. Hemodynamic studies have further shown that FIB-4 correlates with elevated right-sided filling pressures and markers of congestion, suggesting its association with right ventricular dysfunction due to left-sided HF [25,31]. Similarly in patients with heart failure with preserved ejection fraction (HFpEF), an increase in the FIB-4 index was associated with right ventricular dysfunction (assessed by echo) and a higher risk of future MACE, defined as the composite of cardiovascular death, readmission for heart failure, nonfatal myocardial infarction, and nonfatal stroke. During a median follow-up of 736 days, 37 MACE events occurred. Multivariate Cox regression analysis revealed that a high FIB-4 index before discharge (per 1 point) was a significant predictor of MACE (hazard ratio 1.270, 95% CI 1.052–1.532). Receiver operating characteristic analysis indicated that the optimal cut-off value of FIB-4 index before discharge to predict MACE was 3.11. Patients with an FIB-4 index before discharge ≥ 3.11 had a significantly poorer prognosis than patients with FIB-4 index before discharge < 3.11 (*p* = 0.029). Patients with an FIB-4 index ≥ 3.11 had a 2.202-fold (95% CI 1.110–4.368) increased risk of MACE compared with those with an FIB-4 index < 3.11 [32]. These findings support the notion that in HF populations, FIB-4 reflects not only underlying hepatic fibrosis but also the severity of systemic congestion and cardio-hepatic interplay that arise from elevated venous pressures and reduced cardiac output.

### 4.2. FIB-4 in Tricuspid Regurgitation and Pulmonary Arterial Hypertension

Emerging evidence suggests that the FIB-4 index may have prognostic relevance in significant tricuspid regurgitation (TR). In a recent large retrospective cohort of patients with atrial fibrillation, higher FIB-4 values correlated with the severity of TR and were independently associated with increased all-cause mortality over nearly four years of follow-up [33]. Additionally, in a dual-center study of patients with severe isolated TR, elevated FIB-4 scores were significantly associated with an increased incidence of major adverse cardiovascular events, including death and HF hospitalization [34]. Emerging evidence suggests that liver dysfunction and fibrosis-related pathways may reflect systemic congestion, inflammation, and adverse hemodynamics associated with PH. Direct evaluations of the FIB-4 index in PH populations, including CTEPH, are more limited but offer valuable insight into potential mechanisms. In patients with pulmonary hypertension due to left heart disease (PH-LHD) confirmed by RHC, the FIB-4 index has been identified as a predictor of all-cause mortality, suggesting that elevated venous pressures and resultant hepatic dysfunction evaluated by FIB-4 have prognostic relevance even in post-capillary PH phenotypes [35].

In PAH patients, single-center cohorts (61 patients diagnosed with PAH, follow-up 27 months) have shown that higher FIB-4 values are associated with worse survival, with ROC-based thresholds demonstrating reasonable discrimination for mortality risk. During the subsequent evaluation of the study cohort, 20 patients were found to have experienced mortality. The group with mortality had higher FIB-4 scores (1.6 ± 0.22 vs. 0.69 ± 0.4, *p* = 0.003). Independent predictors of mortality and the diagnostic performance of the FIB-4 index were analysed by ROC curve analysis. Accordingly, the predictive value of the FIB-4 index for mortality was >1.02, with a sensitivity of 77% and specificity of 72% [36].

Nevertheless, the current body of literature lacks systematic reviews and robust analyses specifically evaluating the role of FIB-4 in CTEPH. This notable gap in the literature supports the hypothesis that FIB-4 may serve as a clinically accessible marker reflecting disease severity and prognosis in patients with CTEPH.

### 4.3. Improvement After Balloon Pulmonary Angioplasty

We hypothesized that BPA, by reducing pulmonary vascular resistance and improving right ventricular function, alleviates hepatic congestion and systemic hemodynamic impairment, resulting in a measurable reduction in FIB-4 values. Demonstrating such an effect would support the concept that a reduction in FIB-4 reflects hemodynamic and functional improvement following interventional therapy in CTEPH and may serve as a surrogate marker of treatment response in this population.

The reduction in FIB-4 observed after BPA should not be interpreted as evidence of regression of established hepatic fibrosis or as a direct measure of improvement in liver function. Rather, it represents a change in a composite non-invasive index that may be influenced by cardiohepatic congestion and hemodynamic status. In chronic thromboembolic pulmonary hypertension, elevated FIB-4 values may be driven by right heart failure–associated hepatic congestion, manifested by alterations in aminotransferase levels and platelet count, rather than by permanent fibrotic liver disease.

First, BPA has been shown to significantly improve pulmonary hemodynamics by reducing mean pulmonary arterial pressure and pulmonary vascular resistance, while improving right ventricular function and exercise capacity [4,5,8,10,12]. Enhanced pulmonary and systemic blood flow following BPA may lead to improved hepatic perfusion and reduced venous congestion.

Second, patients with CTEPH are characterized by chronic systemic inflammation and endothelial dysfunction [37,38,39], which are reflected by elevated levels of inflammatory mediators, including interleukin-6, interleukin-8, and endothelin-1 [38,39,40]. Previous studies have demonstrated a reduction in these inflammatory markers after BPA [39,41,42]. Attenuation of systemic inflammation may, in turn, influence platelet count and liver-related laboratory parameters, which constitute key components of the FIB-4 index [15,39].

Importantly, among the individual components of FIB-4, platelet count increased significantly after BPA, whereas AST and ALT did not change significantly. A post hoc mathematical decomposition confirmed that the change in platelet count was the largest contributor to the observed change in FIB-4. Although platelet count is an integral component of FIB-4 and was associated with biopsy-defined fibrosis in the original derivation study by Sterling et al. [30], in congestive hepatopathy it may also reflect portal hypertension and right-sided hemodynamic abnormalities. Thus, the increase in platelet count after BPA may reflect improvement in the congestion-related component of cardiohepatic dysfunction rather than regression of structural hepatic fibrosis.

Finally, the combined effects of improved hemodynamics and a reduced inflammatory burden may limit chronic hepatic stress and platelet consumption, thereby contributing to a decrease in the FIB-4 index. Although FIB-4 has not yet been systematically evaluated in patients undergoing BPA, our findings suggest that this non-invasive index may reflect changes in cardiohepatic congestion and systemic hemodynamics following intervention.

In an exploratory multivariable linear regression analysis, change in NT-proBNP was associated with concurrent change in FIB-4. This finding may indicate that changes in FIB-4 occur in parallel with the overall cardiac response to BPA but should be regarded as hypothesis-generating.

NT-proBNP integrates right ventricular wall stress and overall circulatory burden. This observation is biologically plausible, as relief of venous congestion and improved cardiac output have been shown to ameliorate congestive hepatopathy. The lack of independent associations with other clinical or hemodynamic variables suggests that NT-proBNP serves as a robust surrogate marker of the global hemodynamic response to BPA [43]. Importantly, reductions in FIB-4 should be interpreted as reflecting improved hemodynamics and systemic perfusion rather than regression of structural hepatic fibrosis [18,19,22,44].

Notably, a reduction in FIB-4 was confined to a subset of patients, indicating heterogeneity in the contribution of congestion-related hepatic dysfunction to baseline FIB-4 elevation. These findings underscore the need for cautious interpretation of non-invasive fibrosis scores in patients with pulmonary hypertension and right heart failure, particularly when used to infer structural liver disease.

## 5. Conclusions

In our study, the FIB-4 index was elevated in 77.4% of patients with CTEPH. BPA is an effective procedure that markedly improves hemodynamics and alleviates right heart failure. In addition, an improvement in the FIB-4 category was observed in approximately one-third of patients with elevated baseline FIB-4, while most patients remained in the same category. A reduction in NT-proBNP, a marker of right ventricular dysfunction, was the only variable associated with the concurrent change in FIB-4 index. These findings indicate that FIB-4 decreases after BPA in a subset of patients and that its change is associated with the hemodynamic response to treatment; however, whether this represents a clinically meaningful hepatic benefit remains uncertain. FIB-4 may serve as an accessible marker reflecting right-sided hemodynamic burden and systemic congestion, but its clinical utility in CTEPH should be evaluated in prospective studies assessing its incremental value and responsiveness to disease-modifying interventions.

### 5.1. Clinical Implication

In patients with chronic thromboembolic pulmonary hypertension, changes in FIB-4 after BPA should be interpreted cautiously as changes in a composite non-invasive index occurring alongside improvement in right-heart hemodynamics, rather than as evidence of regression of structural liver fibrosis.

Future studies are warranted to prospectively evaluate changes in the FIB-4 index after BPA and to clarify its potential role as a marker of metabolic and hemodynamic response in patients with CTEPH.

### 5.2. Study Limitations

This study has several limitations. First, its retrospective and single-centre design may limit the completeness of available clinical data and the generalizability of the findings and precludes causal inference. Second, the relatively small sample size reduces statistical precision and limits the robustness of the exploratory multivariable regression analysis. Third, the absence of a control group prevents us from determining whether the observed changes in FIB-4 were specifically attributable to BPA. Finally, the study focused on a single non-invasive composite index, FIB-4, whose conventional cut-offs may have reduced specificity and increased false-positive classifications in older individuals due to the incorporation of age into the formula. Therefore, our findings should be considered exploratory and hypothesis-generating. Larger prospective studies incorporating direct assessments of liver fibrosis and hepatic congestion are needed to clarify the clinical significance of changes in FIB-4 following BPA.

## Figures and Tables

**Table 1:** **Clinical characteristic of the study group.** BMI—body mass index, AF—atrial fibrillation, AFL—atrial flutter; BPA—balloon pulmonary angioplasty.

Variable:	
Age (years)	68 (58–73)
Females (n, %)	13 (41.9)
BMI (kg/m^2^)	25.8 (23.1–30.1)
**CTEPH-related thromboembolic medical history**	
Prior pulmonary embolism (n, %)	25 (80.6%)
Prior deep venous thrombosis (n, %)	17 (54.8%)
Inferior vena cava filter (n, %)	3 (9.7%)
Hypothyroidism (n, %)	1 (3.2%)
History of cancer (n, %)	6 (19.4%)
Splenectomy (n, %)	0
Chronic inflammatory diseases (n, %)	7 (22.6%)
Thrombophilia (n, %)	5 (16.1%)
Antiphospholipid syndrome (n, %)	1 (3.2%)
**Comorbidities and other relevant medical history**	
Arterial hypertension (n, %)	17 (54.8%)
Chronic kidney disease (n, %)	9 (29%)
Coronary artery disease (n, %)	8 (25.8%)
AF/AFL (n, %)	9 (29%)
Diabetes mellitus (n, %)	12 (38.7%)
Dyslipidaemia (n, %)	20 (64.5%)
Chronic obstructive pulmonary disease (n, %)	5 (16.1%)
Prior COVID-19 infection (n, %)	12 (38.7%)
Smoking (n, %)	10 (32.3%)
Prior stroke (n, %)	3 (9.7%)
Chronic heart failure (n, %)	8 (25.8%)
Psychiatric disorders (n, %)	4 (12.9%)
History of exacerbation of right ventricular failure (n, %)	8 (25.8%)
**Background therapy**	
Sildenafil (n, %)	1 (3.2%)
Riociguat (n, %)	28 (90.3%)
Chronically on oxygen (n, %)	4 (12.9%)
**BPA (number of procedures)**	94
Number of sessions (median, IQR)	3 (2–4)
Number of lesions treated (median, IQR)	17 (10–24)

**Table 2:** **Effects of BPA on various parameters.** BPA—balloon pulmonary angioplasty, WHO-FC—World Health Organization Functional Classification. MRA—mineralocorticoid-receptor antagonist. SGLT2—Sodium-Glucose Linked Transporter 2. ACE-I—angiotensin-converting enzyme inhibitors. ARNI—angiotensin receptor-neprilysin inhibitors.

	Before BPA	Post BPA	*p*
Clinical parameters			
WHO-FC			
I	0	10 (32.3%)	<0.0001
II	3 (9.7%)	18 (58.1%)	
III	28 (90.3%)	3 (9.7%)	
IV	0	0	
Heart rate (bpm)	75 (66–85)	74 (65–80)	0.18
6 min walking test distance (m)	380 (260–408)	440 (352–526)	<0.0001
Hemodynamics			
Mean right atrial pressure (mmHg)	8 (5–10)	4 (3–5)	<0.0001
Mean pulmonary artery pressure (mmHg)	42 (33–50)	33 (30–39)	<0.0001
Cardiac index (L/min/m^2^)	2.3 (1.9–2.7)	2.6 (2.3–3)	0.008
Pulmonary artery wedge pressure (mmHg)	9 (6–12)	9 (7–12)	0.24
Stroke volume index (ml/m^2^)	29.8 (23.2–36.1)	33.8 (27–39.8)	0.009
Pulmonary vascular resistance (Wood Units)	8.2 (5.3–9.7)	4.8 (4.2–6.8)	<0.0001
Mixed venous blood saturation (%)	66 (63–68)	69 (67–70)	<0.0001
Echocardiography			
TAPSE/sPAP	0.2 (0.17–0.25)	0.34 (0.3–0.5)	<0.0001
Laboratory parameters			
NT-proBNP (pg/mL)	803 (344–2060)	367 (121–866)	<0.0001
Creatinine µmol/L	89 (73–107)	92 (77–106)	1
ALT (U/L)	23 (17–32)	21 (15–34)	0.65
AST (U/L)	27 (23–34)	26 (18–34)	0.24
Hb (g/dL)	14 (13.2–15)	13.5 (13–14.7)	0.44
PLT (×10^3^/L)	191 (144–266)	220 (180–309)	0.01
FIB-4	1.94 (1.34–2.9)	1.65 (0.97–2.22)	<0.0001
Medications			
Loop diuretics (n, %)	23 (74.2%)	19 (61.3%)	0.13
MRA (n, %)	21 (67.7%)	19 (61.3%)	0.69
SGLT2 inhibitors (n, %)	4 (12.9%)	7 (22.6%)	0.25
ACE-I/ARNI (n, %)	10 (32.3%)	6 (19.4%)	0.013
Statins (n, %)	14 (45.2%)	21 (67.7%)	0.02
V1a/V2 receptor antagonists (n, %)	0	0	1

## Data Availability

The data supporting the findings of this study are available from the corresponding author upon reasonable request.
